# Method to Prevent the Springing of the Plate during the Process of Soldering on the Teeth

**Published:** 1849-07

**Authors:** 


### METHOD TO PREVENT THE SPRINGING OF THE PLATE DURING THE PROCESS OF SOLDERING ON THE TEETH.

My Brother, E. Taylor, some time since, adopted the following plan, which so far has answered a good purpose: "Take an iron wire, the size of a knitting needle, bend it double, the strands one-fourth of an inch apart; take a smaller wire, and fold on the other back and forward, so as to make a net work. The whole when completed, should be the length of the labial surface of the teeth, and curved to correspond with the job to be soldered. When the job is put into the plaster and sand, and we use for this a cast iron box, the net work of wire is imbedded in the plaster and sand, outside of the teeth, and between them and the border of the cast iron box. This net work of wire is filled thus with the plaster, and in heating, holds the plaster and sand together, so that the heat requisite for the process of soldering, will not crack it; an additional wire might thus be placed in the concave surface of the plate, and thus give additional security to the plate." The profession will please test this, and if they know of anything better, give it publicity. —[Cin. Ed.

